# Inhalation of Molecular Hydrogen, a Rescue Treatment for Noise-Induced Hearing Loss

**DOI:** 10.3389/fncel.2021.658662

**Published:** 2021-06-01

**Authors:** Anette Elisabeth Fransson, Pernilla Videhult Pierre, Mårten Risling, Göran Frans Emanuel Laurell

**Affiliations:** ^1^Department of Surgical Sciences, Uppsala University, Uppsala, Sweden; ^2^Department of Neuroscience, Karolinska Institutet, Stockholm, Sweden; ^3^Department of Clinical Science, Intervention and Technology, Karolinska Institutet, Stockholm, Sweden

**Keywords:** continuous broadband noise, synaptophysin, densitometry, protective effects of hydrogen, outer hair cells, auditory brainstem response

## Abstract

Noise exposure is the most important external factor causing acquired hearing loss in humans, and it is strongly associated with the production of reactive oxygen species (ROS) in the cochlea. Several studies reported that the administration of various compounds with antioxidant effects can treat oxidative stress-induced hearing loss. However, traditional systemic drug administration to the human inner ear is problematic and has not been successful in a clinical setting. Thus, there is an urgent need to develop rescue treatment for patients with acute acoustic injuries. Hydrogen gas has antioxidant effects, rapid distribution, and distributes systemically after inhalation.The purpose of this study was to determine the protective efficacy of a single dose of molecular hydrogen (H_2_) on cochlear structures. Guinea pigs were divided into six groups and sacrificed immediately after or at 1 or 2 weeks. The animals were exposed to broadband noise for 2 h directly followed by 1-h inhalation of 2% H_2_ or room air. Electrophysiological hearing thresholds using frequency-specific auditory brainstem response (ABR) were measured prior to noise exposure and before sacrifice. ABR thresholds were significantly lower in H_2_-treated animals at 2 weeks after exposure, with significant preservation of outer hair cells in the entire cochlea. Quantification of synaptophysin immunoreactivity revealed that H_2_ inhalation protected the cochlear inner hair cell synaptic structures containing synaptophysin. The inflammatory response was greater in the stria vascularis, showing increased Iba1 due to H_2_ inhalation.Repeated administration of H_2_ inhalation may further improve the therapeutic effect. This animal model does not reproduce conditions in humans, highlighting the need for additional real-life studies in humans.

## Introduction

Worldwide, there is growing interest in pharmacological treatments to protect the inner ear during harmful exposure and treat inner ear disorders. To date, despite numerous studies, there are no effective treatments for acute acoustic injury.

Noise is a major environmental factor causing acquired hearing loss. Because the human cochlea is the deepest part of the ear, localized in the temporal bone, it is a hidden structure inaccessible to detailed clinical assessments. Therefore, most of our knowledge of acquired hearing loss derives from preclinical studies. The organ of Corti in the cochlea contains two types of hair cells, one row of inner hair cells (IHCs) and three rows of outer hair cells (OHCs). The IHCs are sensory receptor cells, and the OHCs serve as amplifiers of inner ear mechanics. Noise-induced hearing loss (NIHL) results from oxidative stress, mitochondrial damage, and excessive glutamate release at the synapse between the IHC and its afferent neuron, the inner hair cell ribbon synapse (Pujol and Puel, 1999; Hu et al., 2002). Elevated formation of ROS and free radicals is considered an important factor in producing OHC loss and is seen as a key factor for pharmacological prevention and treatment of NIHL (Yamashita et al., 2004; Rybak et al., 2019). ROS are overproduced during intense noise in various cochlear cells, including OHCs, blood vessel cells, supporting cells, and spiral ganglion neurons (Mattson, 2000; Yamashita et al., 2004; Henderson et al., 2006). There may be a prolonged overexpression of ROS in cochlear tissue after acoustic overstimulation that persists for 2 weeks (Mattson, 2000; Yamashita et al., 2004; Henderson et al., 2006). Immune reactions also follow acoustic overstimulation which can initially be seen as the release of proinflammatory cytokines and stimulation of innate receptors found in the macrophages/microglia (Wakabayashi et al., 2010; Vethanayagam et al., 2016). The inflammatory process involves recruiting immune cells of both the innate and adaptive systems (Hirose et al., 2005; Wakabayashi et al., 2010; Wood and Zuo, 2017).

One major problem in pharmacological treatments of inner ear disorders is that the barrier systems in the cochlea prevent medications in systemic circulation from gaining access to the deeper cochlear compartments and, in particular, to the organ of Corti. The stria vascularis in the cochlea’s lateral wall blocks certain compounds and molecules from reaching the organ of Corti; nevertheless, some respired gases diffuse into the deepest compartment of the cochlea and may therefore serve as therapeutic agents (Shi, 2016). Recent work in animal models demonstrated that molecular hydrogen (H_2_) might treat oxidative stress-induced hearing loss because hydrogen potentially penetrates through the cochlear barriers and reaches the organ of Corti. H_2_ has anti-antioxidant and anti-inflammatory properties (Gharib et al., 2001; Fukuda et al., 2007; Xie et al., 2010); it reduces the hydroxyl radical (·OH) to H_2_O, prevents lipid peroxidation and DNA damage, and stabilizes the mitochondria (Ohsawa et al., 2007). Animal models showed that hydrogen delivered in various ways induced therapeutic effects. Preclinical studies used intraperitoneal injections of hydrogen-saturated saline (Zhou et al., 2012; Chen et al., 2014), oral intake of hydrogen-saturated water (Zhang et al., 2012), and inhalation of gaseous H_2_ (Fukuda et al., 2007; Huang et al., 2010; Kurioka et al., 2014). Inhalation of H_2_ also prevented hearing loss caused by the antineoplastic drug cisplatin (Fransson et al., 2017).

Based on these findings, we designed the following study to investigate the longitudinal effects of H_2_ inhaled immediately after noise exposure.

## Materials and Methods

### Experimental Design

Freely moving guinea pigs with normal hearing were exposed to broadband noise (2–20 kHz) for 2 h followed by inhalation of H_2_ or air for 1 h. The animals were randomly divided into the following six groups; group Acute + H_2_ (*n* = 8) and group Acute + Air (*n* = 8) were sacrificed immediately after inhalation of H_2_ and air, respectively; group 1w + H_2_ (*n* = 8) and group 1w + Air (*n* = 8) were sacrificed 1 week after inhalation of H_2_ and air, respectively; and group 2w + H_2_ (*n* = 8) and 2w + Air (*n* = 8) were sacrificed 2 weeks after inhalation of H_2_ and air, respectively. All animals were tested for normal hearing prior to the noise exposure and before sacrifice with frequency-specific acoustic auditory brainstem response (ABR). We performed morphological and immunohistochemical analyses, specifically assessing hair cell loss and changes to the IHC and OHC synapse region and immune responses. Synaptophysin, a glycoprotein marker of synaptic membrane and small synaptic vesicles, was used to determine the space under the basolateral membrane of the IHCs and the OHCs corresponding to the site where nerve fibers make synaptic contact with the hair cells. Synaptophysin is reported to be found in the axosomatic efferent synapses under the IHCs and OHCs (Counter et al., 1997; Park et al., 2011). The distribution of Iba1, a calcium-binding adaptor molecule that binds to macrophages/microglia was assessed to analyze the immune response in the stria vascularis. An additional five animals not exposed to noise were used only in the immunohistochemistry part of the study.

### Animals

A total of 53 albino guinea pigs of both sexes (262–337 g; Harlan Laboratories Inc., The Netherlands) were used. The animals were kept in an enriched environment and housed with a 12/12 h day and night cycle with free access to food and water at a temperature of 21°C and a humidity of 60%. All animal procedures were performed in accordance with the ethical guidelines of Uppsala University and consistent with the national regulations for care and use of animals. The local ethics committee approved the experimental procedures (Uppsala ethical committee on animal experiments; approval C 74/16).

### Auditory Brainstem Response (ABR)

The animals were deeply anesthetized using xylazine (10 mg/kg, intramuscularly; Bayer, Denmark) and ketamine (40 mg/kg, intramuscularly; Pfizer AB, Sweden). Ophthalmic ointment was applied to the eyes to prevent corneal ulcers resulting from lack of blink reflex due to ketamine. The animals were placed in a soundproof box and frequency-specific ABRs at 3.15, 6.3, 12.5, and 20 kHz were recorded to monitor the auditory function. The frequency-specific electrohysiological thresholds were determined at two time points for each animal, before noise exposure and at day 1(Acute), 1 or 2 weeks, respectively. A signal analyzer generated the stimulus signals (Tucker-Davies Technologies, FL, USA) controlled by a personal computer and presented through an electrostatic speaker (EC1; Tucker-Davies Technologies, FL, USA). The speaker was connected to an 8-cm tube placed in the guinea pigs’ ear canal. Neural responses were collected using three subdermal electrodes placed at the vertex (active), the mastoid (reference), and in the hind leg (ground). We defined the ABR threshold as the lowest stimulus intensity that produced a reproducible response for ABR wave II observed at the same latency after an average of 1,000 recordings.

### Noise

Two guinea pigs were simultaneously exposed in individual cages, placed side by side inside a soundproof box. The noise, generated by Brüel and Kjær 3560-C PULSE hardware and a LAB 300 amplifier with PULSE LabShop version 13.1.0.246 software (Brüel and Kjær, Denmark), was presented through a transducer (Model 2482, JBL, LA, USA) with a Beyma TD-360 horn (Acustica Beyma, Spain) centrally positioned inside the box 75 cm above the animal cages. The noise was calibrated with a microphone (Brüel and Kjær, Denmark) and adjusted to the desired level before each exposure. The guinea pigs were exposed to a broadband free-field noise; 2–20 kHz for 2 h at 115 ± 2 dB SPL.

### H_2_ Administration

A gas mixture of H_2_ (2 mol%), oxygen (O_2_; 21 mol%), and nitrogen (N_2_; 77 mol%; AGA Gas AB, Sweden) was used. After noise exposure, the animal was anesthetized as previously described (Fransson et al., 2017) and the gas was administered over 1 h through a facial mask with a flow rate at 0.5 L/min using a single-stage pressure regulator (C 200/1A B 3 BAR DIN1, Linde Gases Division, Germany).

### Morphology

After the second ABR measurement, the animal was decapitated. The temporal bones were removed, and the bullae were opened to expose the cochleae. We created small fenestrations in the apex and the round window (RW) within minutes after decapitation and gently flushed 4% phosphate-buffered formaldehyde through the cochlea. The left ear was used for surface preparation and the right ear for cryosectioning. As previously described, we performed surface preparation and hair cell counting (Canlon and Fransson, 1995). Briefly, we removed the bone and tissues surrounding the organ of Corti and rinsed the tissue in phosphate-buffered saline (PBS), placed them in a 0.3% Triton-X100 solution for 10 min, rinsed and incubated with fluorescent-labeled phalloidin (FITC 1:200, Sigma–Aldrich) for 45 min followed by multiple rinses. The organ of Corti was dissected and mounted in glycerol on a glass slide, covered with a coverslip. All OHCs and IHCs throughout the cochlea were examined using a Zeiss Axio Observer.Z1 microscope with a 40x objective for all OHCs and IHCs throughout the cochlea. After analyzing and counting all hair cells and scar formations, we calculated the percentages of hair cell loss per millimeter and plotted them in a cochleogram. To present average data, we divided the basilar membrane into three parts calculated from the round window (RW) in mm, apex (18–14.1 mm from RW), middle (14–9.1 mm from RW), and base (9–2.1 mm from RW).

The right ear was decalcified in 0.1 M EDTA, rinsed and placed in 15% sucrose for 24 h followed by a gradual infiltration of 15% sucrose and OCT Cryomount (Histolab, Sweden) for 4 days ending with only OCT overnight before being embedded in OCT. The cochleae were cryosectioned at 10-μm thickness throughout the cochlea and mounted on SuperFrost Plus slides (Menzel-Gläser, Braunschweig, Germany).

### Immunohistochemistry and Densitometry Measurement

To investigate the presence of synaptophysin and Iba1 in the cochlea, we performed immuno-histochemistry labeling. As primary antibodies, we used monoclonal anti-synaptophysin (mouse IgG1 isotype) diluted 1:200 (Sigma–Aldrich, Inc., MO, USA) and polyclonal Iba1 (rabbit IgG) diluted 1:300 (Thermo Fisher Scientific, IL, USA).

All slides were placed at room temperature for 30 min followed by 15 min in 0.01 M PBS. Primary antibodies were diluted in a solution of 0.3% Triton, 5% bovine serum albumin, 5% normal donkey serum, and 0.1% sodium azide in 0.01 M PBS. The slides were incubated with the primary antibody overnight in 4°C. The following day the slides were rinsed thrice in 0.01 M PBS (5 min/wash), followed by 1 h incubation with the secondary antibodies, which was DyLight 488 (Jackson ImmunoResearch, USA) for synaptophysin, and DyLight 594 (Jackson ImmunoResearch, USA) for Iba1. Both secondary antibodies were diluted 1:400 with 0.01 M PBS, 0.3% Triton, and 0.1% sodium azide. After the final rinse (three rinses for 10 min each), a coverslip was mounted using Mowiol mounting medium (Sigma–Aldrich Inc.).

The quantification of synaptophysin and Iba1 was performed by densitometry measurement using ImageJ, 1.51j8 software (National Institutes of Health, Bethesda, MD, USA; Schneider et al., 2012). Using the software ImageJ, the intensity of the immunofluorescent staining was objectively measured and processed.

**Figure 1:**
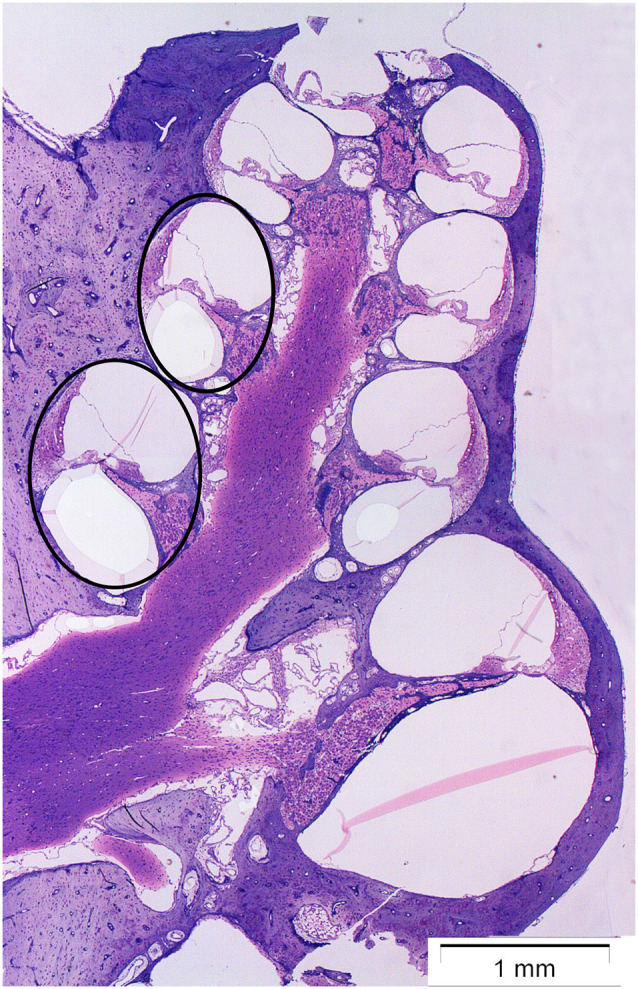
Micrograph showing a midmodiolar cross-section of the normal guinea pig cochlea. The black circles indicate the two points on the basilar membrane 12 mm (middle turn) and 7 mm (basal turn) from the round window, where the immunoreactivity of synaptophysin, and Iba1 was analyzed.

The cochlea was analyzed at two points on the basilar membrane, 7 and 12 mm from the RW (Figure 1) which correlate tonotopically to the frequencies 12.5 kHz and 3.15 kHz, respectively (von Békésy, 1960). All sections were stained simultaneously and all images were acquired within a few days after staining.

The method for the quantification of the densitometry measurement has previously been described in detail (Fransson et al., 2017). Briefly, we collected and processed images using the ImageJ software. The software converts the color to a grayscale and measures pixel intensity ranging from black (0) to white (255). These counts were used to quantify the fluorescent intensity of the regions of interest (ROIs) and the values obtained from these measurements are referred to as intensity. The outline of the ROIs was manually traced. For the synaptophysin immunoreactivity, the synapse area under the IHCs and the three rows of OHCs were analyzed separately. The ROI for Iba1 was the area of stria vascularis. ImageJ was used to calculate the mean gray value from each ROI.

For each animal, three sections were selected where the regions of 7 and 12 mm from the RW were clearly visible. The images from these sections were analyzed using the densitometry method. The intensity value from each ROI was summed up for each animal. Finally, the mean value for each animal was used to calculate the mean intensity for each group.

### Statistical Analysis

For statistical analysis of the ABR thresholds, hair cell loss, and immunohistochemistry data, one-way ANOVA was conducted followed by *post hoc* analysis using the Holm–Sidak method in Sigma (v 13.0, Systat Inc. USA). We defined statistically significant differences as *p* < 0.05. We expressed the data as mean ± SEM.

## Results

### H_2_ Inhalation Reduces ABR Threshold Shifts and OHC Loss

**Figure 2:**
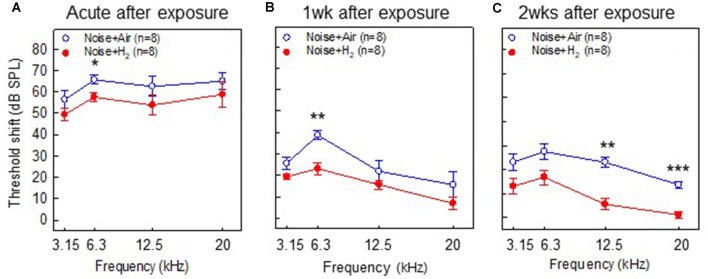
Results from the auditory brainstem response (ABR) measurements performed in **(A)** group Acute + H_2_ and group Acute + Air immediately after inhalation, **(B)** group 1w + H_2_ and 1w + Air 1 week, and **(C)** group 2w + H_2_ and 2w + Air 2 weeks after noise exposure and inhalation of H_2_ or air. The y-axis shows the mean threshold shift as compared to the pretreatment values. Data are presented as mean ± SEM. *p < 0.05, **p < 0.01 and ***p < 0.001.

To test if inhalation of H_2_ administered immediately after noise exposure affected the auditory function, we measured ABR thresholds before the noise trauma and before sacrifice. Compared with the pre-measurements, both the Acute + H_2_ and Acute + Air groups demonstrated significant threshold shifts of approximately 50–60 dB at all four tested frequencies (*p* < 0.001; Figure 2A). There was a significant difference (*p* < 0.05) between the two groups at 6.3 kHz in favor of the H_2_-treated animals. One week after exposure, both the 1w + H_2_ and 1w + Air groups demonstrated significantly smaller ABR threshold shift at all four frequencies (*p* < 0.001) compared to the Acute + H_2_ and Acute + Air groups (Figure 2B). The significant difference between 1w + H_2_ and 1w + Air increased at 6.3 kHz (*p* < 0.01). There were significant differences between 2w + H_2_ and 2w + Air groups at 12.5 (*p* < 0.01) and 20 kHz (*p* < 0.001) in favor of the group treated with H_2._ The 2w + Air group did not recover to normal ABR thresholds at any frequency (Figure 2C). Table 1 displays the differences between ABR thresholds obtained before the noise trauma and at 2 weeks. There was a significant difference across the frequencies except for 20 kHz in the 2w + H_2_ group. Animals treated with H_2_ demonstrated normal hearing threshold sensitivity at 20 kHz. At 12.5 kHz, only a small threshold shift of 6 dB remained (Table 1).

**Table 1:** A comparison of auditory brainstem response (ABR) thresholds obtained at baseline prior to noise exposure and at 2 weeks in the 2w + air and 2w + H_2_ groups.

	Threshold (dB SPL): Pre vs. 2 wk					
	Noise + Air			Noise + H_2_		
	Pre	2 wk		Pre	2 wk	
Freq.	Mean (SEM)	Mean (SEM)	*p*-value	Mean (SEM)	Mean (SEM)	*p*-value
3.15	40.0 (± 2.1)	63.1 (± 3.1)	*p* < 0.001	41.9 (± 1.3)	55.0 (± 2.8)	*p* < 0.001
6.3	23.8 (± 1.2)	51.2 (± 3.6)	*p* < 0.001	25.6 (± 1.5)	43.1 (± 3.0)	*p* < 0.001
12.5	16.2 (± 1.2)	40.0 (± 4.0)	*p* < 0.001	20.0 (± 1.3)	25.6 (± 1.8)	*p* < 0.05
20.0	18.8 (± 1.2)	32.5 (± 2.8)	*p* < 0.001	21.9 (± 1.3)	23.1 (± 1.6)	NS

*No significant threshold shift was found at 20 kHz in H_2_ treated animals. NS, not significant*.

**Figure 3:**
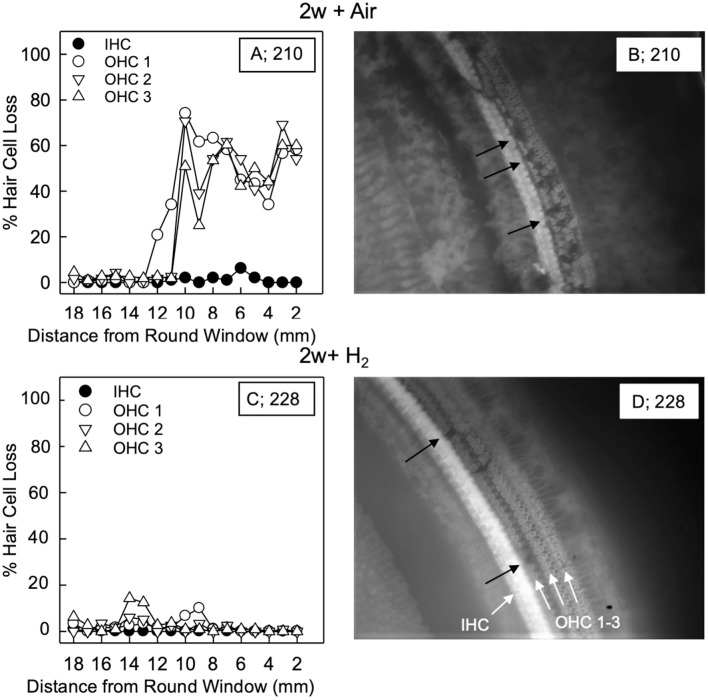
Representative cochleogram from group 2w + Air **(A)** and group 2w + H_2_ **(C)**. **(B)** Micrograph from the surface preparation of the same animal as **(A)** in the 10 mm area from the round window (RW). The black arrows indicate hair cell loss in all three rows of outer hair cells (OHCs). **(D)** Micrograph from the surface preparation of the same animal as **(C)** in the 10 mm area from the RW. The black arrows indicate hair cell loss in the first row of OHCs. The white arrows indicate inner hair cell (IHC) and the three rows of OHCs 1, 2 and 3.

**Figure 4:**
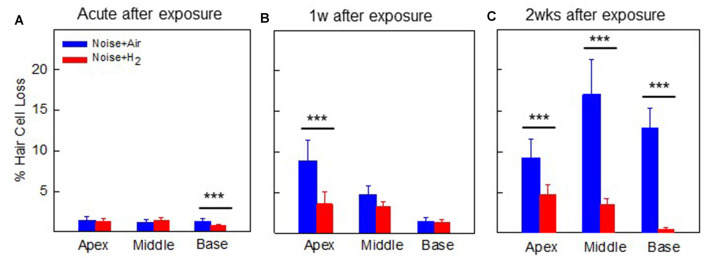
Mean percentage of OHC loss in **(A)** group Acute + H_2_ and group Acute + Air immediately after inhalation, **(B)** group 1w + H_2_ and 1w + Air 1 week, and **(C)** group 2w + H_2_ and 2w + Air 2 weeks after noise exposure and inhalation of H_2_ or Air. Data are presented as mean ± SEM. ***p < 0.001.

For the quantification of hair cell loss, we analyzed both the IHCs and the three rows of OHCs. Figure 3 shows representative cochleograms from groups 2w + H_2_ and 2w + Air. Only a few IHCs were missing in each cochlea, and this applied to all animals regardless of the group (data not shown). The most characteristic finding, loss of OHCs, clearly appeared at 1 and 2 weeks; the ABR thresholds were most severely affected in the two acute groups, and there was only a small percent of OHC loss in these two groups. Interindividual variability was observed in terms of OHC loss. There was a significant difference (*p* < 0.001) in the basal part of the cochlea in favor of the Acute + H_2_ group (Figure 4A). One week after noise exposure, the morphological pattern was different; the amount of OHC loss was more extensive, particularly for the group that inhaled air. There was a significant difference (*p* < 0.001) in the apex area between the 1w + H_2_ and 1w + Air groups (Figure 4B). Two weeks after exposure, the 2w + Air group demonstrated a large and significant (*p* < 0.001) OHC loss in the entire cochlea (Figure 4C).

### Immunohistochemistry

#### Protective Effect of H_2_ on Synaptophysin Immunoreactivity

**Figure 5:**
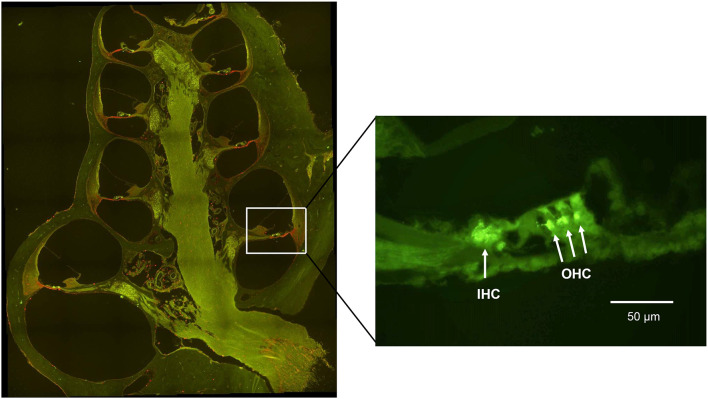
A fluorescent image of a guinea pig cochlea labeled with synaptophysin (green) and Iba1 (red). The enlarged image from the basal turn (7 mm from the RW) shows the synaptophysin immunoreactivity under the IHC and the OHC.

To investigate whether treatment with H_2_-inhalation after noise exposure affected the synapse region’s activity under both the IHCs and OHCs, we performed immunohistochemical staining with synaptophysin, a synaptic intrinsic membrane protein of the synaptic vesicles (Calhoun et al., 1996). We detected synaptophysin immunoreactivity under the IHCs and the OHCs in the organ of Corti (Figure 5). The synapse region under the IHCs was a large, intensive area lacking borders, while the synapse region under each OHC was smaller and more distinct. If an OHC was missing or damaged, we observed no or faint immunoreactivity under the OHC. Two points on the basilar membrane were chosen for quantification of the immunoreactivity, 7 and 12 mm from the RW corresponding to 12.5 kHz and 3.15 kHz, respectively (von Békésy, 1960; Figure 1).

**Figure 6:**
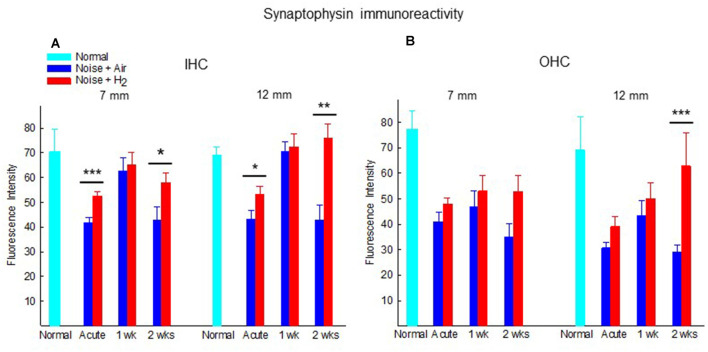
Quantification of the immunoreactivity of synaptophysin in the synapse area of **(A)** IHCs and **(B)** OHCs of animals not exposed (normal) and exposed to noise followed by inhalation of H_2_ (Noise + H_2_) and air (Noise + Air). The immunoreactivity was measured at two points on the basilar membrane, 7 and 12 mm from the round window. Data are presented as mean + SEM. *p < 0.05, **p < 0.01 and ***p < 0.001.

Figure 6 shows the quantification of the immunoreactivity of synaptophysin. In comparison to normal unexposed animals, noise exposure in both acute groups (Acute + H_2_ and Acute + Air groups) induced significant drops of synaptophysin immunoreactivity in the ROIs under IHCs (*p* < 0.05 and *p* < 0.01, respectively, at 7 mm and *p* < 0.05 and *p* < 0.001, respectively, at 12 mm from the RW) and OHCs (*p* < 0.001 for both groups at 7 mm, *p* < 0.05 and *p* < 0.01, respectively, at 12 mm from RW). This is consistent with the severely affected ABR threshold at the first time point assessed after noise exposure. The expression of synaptophysin immunoreactivity in ROIs under the IHCs was significantly stronger in the H_2_ treated groups at the Acute and 2-week time points. We observed this phenomenon both in the basal region (7 mm from the RW) and at 12 mm from the RW, suggesting that inhalation of H_2_ counteracted the deleterious effects of noise on the IHC synaptic structures containing synaptophysin (Figure 6A). Intriguingly, no difference was observed at 1 week. Less pronounced differences between H_2_-treated and air-treated groups were found in the area under the OHCs. The expression of the synaptophysin under the OHCs in the 12 mm area showed a significant difference (*p* < 0.001) between the two groups at 2 weeks after exposure (Figure 6B).

#### Iba1

**Figure 7:**
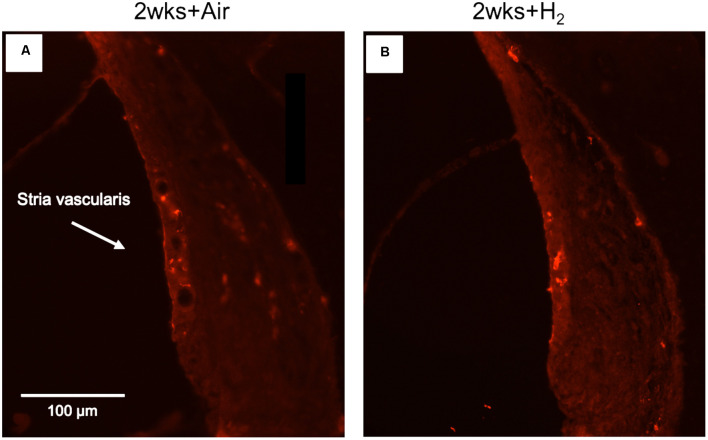
Fluorescent image of Iba1 expression in stria vascularis in high magnification (x40). **(A)** Representative animal from 2w + Air. **(B)** Representative animal from 2w + H_2_.

To investigate whether there were any changes in the expression of macrophages/microglia in the cochlea after noise exposure, the calcium-binding adaptor molecule Iba1 was used as a measure of the immune response. In normal unexposed guinea pigs, Iba1 was found in the basilar membrane, stria vascularis, spiral ligament, Rosenthal’s canal, osseous spiral lamina, and the auditory nerve. The micrograph in Figure 5 shows the Iba1 expression (red) in the inner ear in a representative animal from the 2w + H_2_ group. In Figure 7 Iba1 expression is seen in stria vascularis at high magnification (40×) from an animal in group 2w + Air and an animal in group 2w + H2.

**Figure 8:**
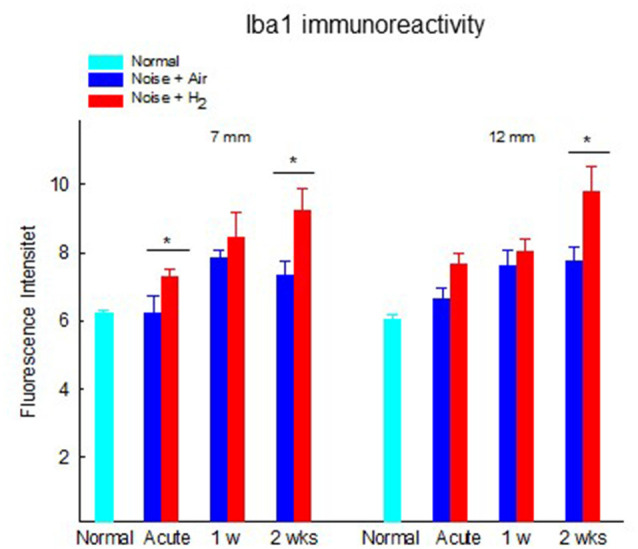
Quantification of the immunoreactivity of Iba1 in the stria vascularis area. The measurements were performed at 2 points corresponding to 7 and 12 mm from the RW. Data are presented as mean + SEM. *p < 0.05.

The results from the quantification of Iba1 expression in the stria vascularis showed a significantly (*p* < 0.01) stronger expression in H_2_-treated animals than in normal-unexposed animals at 7 mm at all three time points. We observed these differences for the air-treated group at 1 and 2 weeks (Figure 8). Iba1 immunoreactivity in stria vascularis at 7 mm was significantly (*p* < 0.05) more intense in the H_2_ treated groups at the acute and 2-week time points, reflecting increased activation of macrophages/microglia in the stria vascularis as a result of H_2_ treatment.

## Discussion

Many of the approaches to protect the cochlea against acoustic overstimulation have shown great promise in experimental models. It is obvious that there are a number of unresolved issues in otoprotection as most of these promising candidates in experimental research have no effectiveness as therapeutic agents in humans. Several reasons may account for this failure such as lack of timely and proper administration of effective drugs. It is also anticipated that the drug must pass the blood-labyrinth barriers to reach the target cells in the cochlea. A growing body of evidence suggests that molecular H_2_ induces therapeutic effects in several medical conditions, mostly in animal models and in small cohorts of patients (Ohsawa et al., 2007; Kang et al., 2011; Chen et al., 2019b). Thus, the physiochemical properties of molecular H_2_ are promising for use in pharmacological treatment of the inner ear.

The search for therapy to intervene with ROS’s cytotoxic effects after acoustic overstimulation is critical. By providing rescue treatment after acoustic trauma at workplaces, hearing could be saved in a substantial number of individuals. In this study, we investigated the otoprotective potential of H_2_, i.e., 2% gaseous H_2_, during 2 weeks in noise-exposed guinea pigs. Anesthetized animals inhaled H_2_ for 1 h starting immediately after 2 h of noise exposure at 115 dB SPL. The guinea pigs were sacrificed at three different time points: immediately, 1 week and 2 weeks after noise exposure followed by H_2_ inhalation in order to follow the electrophysiological and morphological changes at different time points after exposure.

The longitudinal pattern of electrophysiological and morphological alterations differed markedly. While the ABR measurements from the Acute groups showed the most threshold shifts, the cochleograms demonstrated the most significant OHC loss at the final time point 2 weeks later as seen in Figures 2A–C and  4A–C. Inhalation of H_2_ rescued a significant number of OHCs; however, a small percentage loss of OHCs (<5%) remained in the H_2_ treated groups at 1 and 2 weeks. Several factors can explain the high threshold shift seen in the two acute groups, including cochlear ROS overproduction leading to mitochondria dysfunction, mechanical injury to the hair cells and the supportive structures (Hamernik et al., 1984; Kurabi et al., 2017; Ding et al., 2019), and swelling of auditory nerve fibers in the region of their synaptic contact with the IHCs. Swelling of nerve fibers appears immediately after noise exposure and is reported to disappear within a week after noise exposure (Spoendlin, 1971; Robertson, 1983; Kujawa and Liberman, 2015). For the groups of animals that we assessed at 1 and 2 weeks, the better ABR threshold might also reflect the better metabolic status of surviving OHCs, not only in H_2_-treated animals but also in air-treated animals.

Noise-damaged hair cells follow three distinct death pathways: oncotic necrosis, apoptotic cell death, and cell death characterized by no basolateral membrane with cellular debris in an intact OHC (Bohne et al., 2007). Bohne and colleagues reported the time course of OHC degeneration in the chinchilla. It was demonstrated that some OHCs even begin to die during noise exposure, followed by a degeneration process going on for several weeks (Bohne et al., 2017). We did not consider the number of dying hair cells in the morphological analysis because our endpoint measure for the hair cell counting was scar formation. We conclude that a significant ABR threshold shift and reduction of synaptophysin under the IHC appeared in the Acute groups, while the loss of IHCs and OHCs was limited at this time point. These findings corroborate earlier results (Yamashita et al., 2004). Yamashita et al. used a guinea pig model to study the progression of hair cell damage after acoustic overstimulation (Yamashita et al., 2004). The authors found a delayed formation of ROS apical of the main lesion and proposed that this sustained ROS generation correlated with a gradual spread of OHC loss. How the efferent synapses under the IHCs and OHCs react to acoustic overstimulation has been poorly studied earlier. It is reported that the efferent feedback system has a protective effect against noise injury (Liberman et al., 2014). In ageing C57BL/6 mice, degeneration of efferent nerve endings is seen before the loss of IHCs and OHCs (Bartolome et al., 2009). The synaptophysin immunoreactivity was the measure of morphological alteration to the efferent synapses used in the present study. The most consistent finding was a reduced synaptophysin immunoreactivity under IHCs in the acute groups at the two assessed locations along the basilar membrane, 7 mm and 12 mm from the RW. A significant recovery was observed within 1 week in the ROIs under the IHC which was maintained only in the H_2_ treated group at 2 weeks. A more variable pattern was seen in the air treated groups where synaptophysin immunoreactivity after an initial recovery decreased at 2 weeks. Reasons for this variable recovery and loss of synaptophysin immunoreactivity in ROIs under IHCs and OHCs might include delayed formation of ROS and a progressive depletion of vesicles in the axosomatic efferent synapses.

Several earlier studies convincingly showed that noise exposure generates excessive amounts of ROS in the cochlea (Yamashita et al., 2004; Henderson et al., 2006; Böttger and Schacht, 2013). The amount of ROS production depends on the extent of noise exposure. Noise level and duration of exposure are two measures of noise that reflect the amount of acoustic energy hitting the cochlear structures in animals in the laboratory setting. In the present study, we exposed guinea pigs to broadband noise at 115 dB for 2 h in a free field, intending to produce a threshold shift that partly remained after 2 weeks. An interindividual variability of noise-induced changes was found which corroborate earlier findings in the guinea pig (Cody and Robertson, 1983). Inhalation of H_2_ (2 mol%) was used as a rescue treatment. Until recently, little was known about systemic H_2_ distribution. A pharmacokinetic study revealed that inhaled H_2_ dissolves in blood in the lungs and distributes throughout the body by regional blood flow by advection diffusion; then, it undergoes dynamic metabolism (Sano et al., 2020). However, an earlier study showed differences between organs in terms of hydrogen uptake (Yamamoto et al., 2019). Several pharmacological interventions demonstrated the importance of studying NIHL to protect hearing by administration before, during, or after noise exposure (Le et al., 2017; Rybak et al., 2019). However, the accumulation of scientific data through the literature cannot provide answers to whether antioxidant treatment alone can mitigate the noise’s negative impact. NIHL has several targets, and excessive ROS production does not induce all triggered events. The present study’s significant finding is that inhalation of H_2_ has therapeutic effects at several sites in the cochlea. One option would be to provide repeated inhalations for up to 2 weeks after noise exposure to improve therapeutic outcomes.

Earlier studies showed that there are resident macrophages in the human inner ear (O’Malley et al., 2016; Kämpfe Nordström et al., 2018). Acoustic trauma recruits macrophages to the lesion site (Fredelius and Rask-Andersen, 1990; Hirose et al., 2005). Kämpfe Nordström et al. (2018), using super-resolution structured fluorescence microscopy have shown the existence of a large number of Iba1 positive cells with various morphologies in the human inner ear. The results of Iba1 quantification in the present study support the hypothesis that the stria vascularis is one route of H_2_ efflux into the organ of Corti *via* transport through the intrastrial fluid-blood barrier. In the noise-exposed guinea pigs, there was greater Iba1 immunohistochemical expression in the stria vascularis in the H_2_ groups than in normal animals that were more intense immediately after and at 2 weeks in the H_2_ groups than in air-treated groups. These findings suggest that the upregulated Iba1 in the H_2_ groups represent anti-inflammatory macrophages of the M2 type. Because macrophages have several functions, this upregulation indicates that the macrophages promote factors restoring homeostasis after inhalation of H_2_. This phenomenon may especially involve the inner hair cell ribbon synapse, as shown by Kaur et al. (2019). Fujioka et al. (2006) have shown that acoustic trauma produces rapid cochlear production of pro-inflammatory cytokine IL-6 in the lateral wall and spiral ganglion followed by normalization a few hours later. It was suggested that lateral wall fibrocytes produce IL-6 *via* a synergistic interplay with IL-1β and TNFα1. IL-1β is known to be produced by the NLRP3 inflammasome, i.e. cytosolic multiprotein complexes that are important mediators of immune response (Kelley et al., 2019). A mouse sepsis model study showed that H_2_ inactivates NLRP3 inflammasome and mitochondrial dysfunction; this may represent the impaired release of proinflammatory cytokines (Chen et al., 2019a). There is also evidence that prolonged intraperitoneal administration of hydrogen-saturated saline before noise exposure reduces levels IL-1, Il-6, and TNFα in the cochlea after noise exposure (Chen et al., 2017). Indeed, it may well be that molecular H_2_ act as an NLRP3 inflammasome inactivator in the inner. These findings and our results indicate that molecular H_2_ promotes OHC survival *via* multiple mechanisms, not only *via* an antioxidative effect but also by modulation of the immune response. The metabolic effects of acoustic trauma on the cochlea were otherwise studied in the context of oxidative stress, especially mitochondrial ROS (Böttger and Schacht, 2013). In parallel to oxidative stress, acoustic overstimulation increased the OHC cytosolic calcium concentration leading to mitochondrial calcium overload, initiating cell death pathways (Fridberger et al., 1998; Wang et al., 2018). It is unknown how these two pathophysiological events interact in the OHC mitochondria and trigger cell death.

Taken together, our findings suggest that inhalation of molecular H_2_ immediately after noise exposure can counteract pathological changes induced by acoustic overstimulation.

## Conclusions

One-hour H_2_ inhalation protected guinea pigs’ ears from noise-related damage immediately after exposure to continuous noise for 2 h. There was a significant reduction of electrophysiological hearing thresholds and OHC loss 2 weeks after noise exposure. H_2_ also preserved synaptophysin immunostaining. Immune reactions induced by noise activated macrophage/microglia cells and H_2_ intensified these effects.

## Data Availability Statement

The raw data supporting the conclusions of this article will be made available by the authors, without undue reservation.

## Ethics Statement

The animal study was reviewed and approved by Uppsala ethical committee on animal experiments; approval C74/16.

## Author Contributions

AF and GL designed the study. AF collected all data, conducted the analyses, and wrote the first manuscript. GL contributed to analyses and the final manuscript. PVP and MR provided conceptual input and contributed to the final manuscript. All authors contributed to the article and approved the submitted version.

## Conflict of Interest

The authors declare that the research was conducted in the absence of any commercial or financial relationships that could be construed as a potential conflict of interest.
